# Valorization of Olive Tree Pruning and By-Products from the Truck Industry in the Manufacture of Low-Environmental-Impact Particleboard

**DOI:** 10.3390/ma18143258

**Published:** 2025-07-10

**Authors:** Juan José Valenzuela Expósito, Elena Picazo Camilo, Griselda Elisabeth Perea Toledo, Francisco Antonio Corpas Iglesias

**Affiliations:** Higher Polytechnic School of Linares, University of Jaén, 23700 Linares, Spain; epicazo@ujaen.es (E.P.C.); gept0001@red.ujaen.es (G.E.P.T.); facorpas@ujaen.es (F.A.C.I.)

**Keywords:** particleboard, core, olive tree pruning, recycled by-products

## Abstract

This study presents the development of particleboards made from olive tree pruning (OTP) residues and truck industry by-products (RCM), using PUR resin as a binder. Five formulations with different OTP/RCM ratios were designed and physical, thermal, mechanical, chemical and microstructural properties were evaluated. The results showed that increasing the RCM content improves the dimensional stability, reduces water absorption and swelling and decreases thermal conductivity, reaching 0.061 W/mK. At the mechanical level, MOR, MOE and IB values of 7.11, 630 and 0.134 MPa, respectively, were obtained. A higher OTP content allows a reduction in the density of the particleboard (752.67 kg/m^3^) due to the granulometry of the material. FTIR and SEM analyses confirmed the good integration of the materials with the resin, highlighting a lower porosity and higher compaction in formulations with a high RCM content. These results demonstrate that the combination of agricultural and industrial by-products is feasible to manufacture a sustainable particleboard with customizable properties, promoting the circular economy and reducing the dependence on virgin raw materials in the construction sector.

## 1. Introduction

The construction sector is highly dependent on conventional raw materials, such as wood, and is currently facing significant environmental challenges. This dependence has caused critical problems associated with a loss of biodiversity through deforestation, the deterioration of ecosystems and increased greenhouse gas emissions [1]. Globally, construction is responsible for 39% of CO_2_ energy emissions and more than half of the raw materials extracted from the environment [2]. Against this backdrop, it is urgent to transform the sector’s practices towards more sustainable models. Growing concern for the environment, together with increasingly demanding environmental regulations, has driven the development and adoption of alternative materials that maintain quality and performance standards and contribute to meeting long-term climate and sustainability goals. For all these reasons, the valorization of waste and industrial by-products is presented as a key strategy to meet the challenges defined in the 2030 Agenda [3].

The expansion of agricultural activity has significantly increased the generation of waste that, in most cases, is not properly managed and accumulates in large volumes and can cause negative environmental impacts [4]. Olive cultivation is widespread in the Mediterranean basin, is a fundamental agricultural activity for the rural economy and is postulated as a source of residual biomass. In countries such as Spain, the world leader in olive grove area and production with 2.70 million hectares of olive groves, crop maintenance tasks, especially annual pruning, generate a large amount of lignocellulosic residues, estimated at 4 Tn of olive tree pruning (OTP) per hectare, which in many cases are not adequately used [5,6]. The selection of OTP as a lignocellulosic raw material in this research is justified both by its abundant availability in olive-growing regions and by its physicochemical characteristics, including its high cellulose and lignin contents, good strength/weight ratio and high capacity for interactions with thermosetting resins. These properties make it competitive with other agricultural residues, while at the same time providing environmental advantages by avoiding its burning or abandonment in the field. OTP residues have physical and chemical characteristics that make them suitable for valorization and reuse as raw materials in the manufacture of different products. Their main application is focused on energy production. However, different authors have studied their use in thermochemical processes such as pyrolysis, gasification or combustion [7,8]. Other studies have focused on their application in the manufacture of products for combustion such as pellets or briquettes [9,10,11] or their application in biorefineries [12,13,14]. Currently, the application as activated carbon in CO_2_ capture is also being investigated [15].

The incorporation of OTP in new products, such as a particleboard, reduces the dependence on virgin raw materials, while contributing to reduce the carbon footprint associated with the production processes [16]. Particleboards are composite materials of a lignocellulosic origin obtained by transforming wood or agricultural waste into particles, which are agglutinated by means of adhesives to form compact panels [17]. One of the main resources used for their manufacture are the by-products of wood processing, such as the remains generated in sawmills, which represent an effective alternative for the production of reconstituted wood derivatives. The production process involves mixing lignocellulosic particles with synthetic adhesives, followed by a hot-pressing stage, during which the adhesive cures and consolidates the panel structure [18,19].

**Figure 1:**
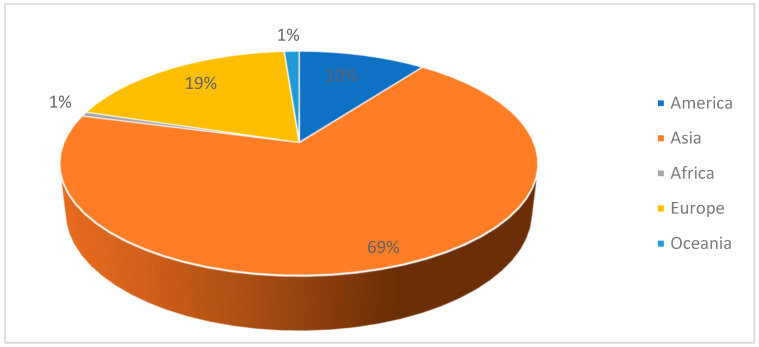
Production quantity of particleboard in the world (m^3^).

**Figure 2:**
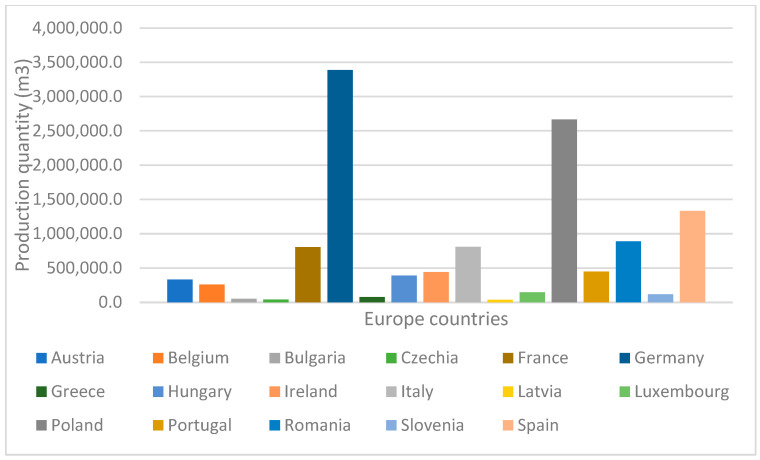
Production quantity of particleboard in Europe (m^3^).

In 2023, particleboard production exceeded 98 million m3 worldwide [20], which is an increase of 2.45% from 2020 (96.01 million) [21]. The largest production of particleboards is located in Asia, with a production volume of 69%, followed by Europe with 19% of the total production (Figure 1). In Europe, Germany, Poland and Spain lead in particleboard production (Figure 2).

The progress made in the development of the particleboard and the potential of this sector as a consumer of waste have led to its application extending beyond the furniture and construction sectors, in the latter, mainly as structural reinforcement, to other applications such as thermal or acoustic insulation, packaging, food packaging or handicrafts [22,23]. The advances experienced in the sector have led to the incorporation of agricultural by-products such as rice, camellia or almond husks and flax, poplar and oak [22,24,25,26] that have demonstrated their viability in the manufacture of particleboards.

As for the adhesives used, there are different types of organic and inorganic binders that allow particles to be bonded together. The most commonly used in the industry are urea-formaldehyde, melamine-urea-formaldehyde and phenol-formaldehyde resins [27,28,29] due to their low cost, ease of application and good adhesive properties. However, the use of these composites also poses environmental and health challenges, especially because of their formaldehyde content [30].

On the other hand, one of the most influential technical parameters on the behavior of the particleboard is its bulk density. This property has a direct impact on the mechanical and hygroscopic characteristics of the material [31,32,33]. Generally, higher densities favor a better compaction of the board, increasing its mechanical strength, although they can also imply a higher weight [34]. In contrast, low-density boards tend to have lower strength values and greater susceptibility to moisture absorption, which may limit their use in certain applications [35,36].

In addition, the composition of the board significantly influences its physical and mechanical performance. The incorporation of alternative materials, such as waste from the car industry composed of polyurethane (PUR), extruded polystyrene (XPS), plywood (PLW) and glass-fiber-reinforced polyester (GFRP), modifies both the internal structure of the panel and its behavior with respect to moisture or mechanical loading. These materials, having different densities, porosities and adhesion capacities, have different effects on the dimensional stability, thermal conductivity and mechanical strength.

Although there is some literature on the application of olive pruning in the manufacture of lignocellulosic particleboards [37], industrially sourced materials such as XPS, PUR or GFRP have been studied mainly in sandwich configurations, structural cores or multilayer elements for non-structural applications [38,39,40,41,42]. So far, no studies have been found that employ these industrial by-products as a reinforcement or filler component within a single lignocellulosic particle-based agglomerated matrix. Therefore, the approach taken in this research is considered to represent an innovative approach in the field of sustainable boards.

Therefore, this research focused on the development of a particleboard using OTP and incorporating by-products from the car industry (PUR, XPS and GFPR) in different fractions, evaluating the effect of these mixtures on the physicochemical, mechanical, microstructural and thermal properties of the final product. The particles were combined with a commercial resin to obtain a quality product that meets the required quality standards. This strategy represents an opportunity to redefine the materials used in particleboard manufacturing and promote sustainability through the application of a circular economy.

## 2. Materials and Methods

### 2.1. Raw Materials

Olive tree pruning (OTP) supplied by Garzón Green Energy (Bailén (Jaén, Spain)) and recycled chopped material (RCM) from waste produced by the truck industry supplied by Liderkit (Guarromán, Jaén (Spain)) were used to manufacture the particleboard.

OTP is generated during the periodic maintenance of olive groves in order to optimize production. Generally, the management of this waste is limited to controlled burning practices or it is abandoned in the olive grove, which generates environmental risks associated with the proliferation of pests [43]. Its composition consists mainly of cellulose, hemicellulose and lignin. Since the OTP used was stored at ambient temperature (21 ± 5 °C), the moisture content ranged between 2 and 3%w. RCM composed of varying proportions of XPS, PUR and GFRP was used as a complementary material in the manufacture of the particleboard. These materials are generated as waste from the cutting of large panels used in the manufacture of truck bodies, which are sectioned to adapt to the dimensions required by the different products manufactured. RCM from vacuum systems was used for the development of this research. Neopur 1791 polyurethane resin supplied by Neoflex (Elche, Spain) together with Adiflex 935 hardener (Neoflex, S.L., Elche, Spain) was used as a binder. Table 1 shows the technical specifications of the resin. Handling times show that during the open period, the resins remain in gel state, which facilitates handling and ensures good workability when mixed at different solid/resin ratios. In the hardening stage, the cores develop between 60 and 70% of their final strength. Finally, the curing time corresponds to the period required to reach between 96 and 98% of the total strength of the system.

**Figure 3:**
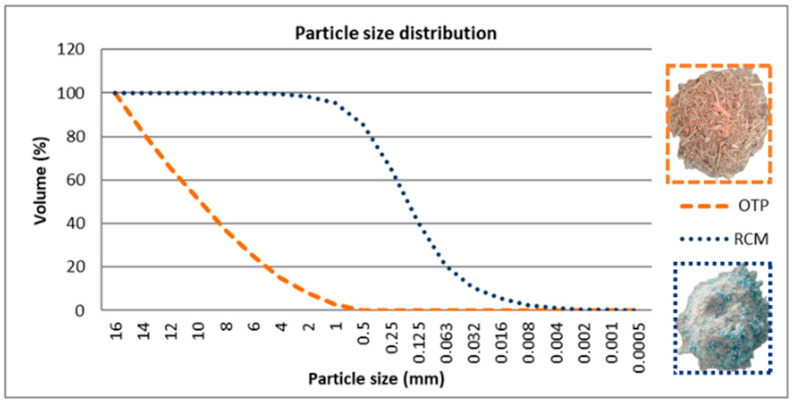
Granulometric distribution of OTP and RCM.

The particle size of OTP and RCM (Figure 3) was determined using Malvern Mastersizer 2000 equipment (Malvern Panalytical, Westborough, MA, USA). The OTP used in this investigation underwent a comminution process in a Viking GE 250 crusher (Stihl, Greenwood, MS, USA) with a 2 cm mesh to reduce its size from between 8 and 12 cm to a particle size of less than 2 cm. After crushing, the OTP was sieved on a 15 mm sieve. The particle size distribution of OTP, conditioned by machining, showed a mean particle size (D_50_) of 9.830 mm and a specific surface area of 1.427 m^2^/g. The waste composing RCM was subjected to a mechanical recycling process in a Felco Europe hammers mill (Felco Europa, S.L., Barcelona, Spain), resulting in a chopped material with a similar particle size. The values of D_50_ and the specific area obtained were 0.151 mm and 159.771 m^2^/g, respectively.

As shown in Figure 3, the particle size distribution curve shows significant differences between the two materials. OTP shows a distribution dominated by large particles, with more than 50% by volume above 2 mm, which is consistent with its fibrous lignocellulosic nature. In contrast, RCM exhibits a fine fraction, with approximately 80% of the particles below 0.25 mm. This particle size implies a high specific surface area, which favors the filling of voids between larger particles, as well as a higher interaction with the resin binder. It should be noted that no additional surface treatment was applied to the OTP particles, beyond drying, in order to preserve their compatibility with the polymer matrix and ensure good interfacial adhesion.

The density of the materials composing the recycled particleboard is essential to define the final mechanical properties. In particular, density directly influences parameters such as mechanical strength and dimensional stability. Higher density is usually associated with a more compact structure, which favors higher mechanical strength. However, this may imply an increase in weight [37,44].

OTP with higher density presents a smaller specific area associated with a more compact structure. The integration of OTP and RCM allows combining structural properties with properties such as thermal insulation or lightness to obtain particleboards with specific properties [38]. OTP presented a density of 427.7 kg/m^3^, which allows to ensure mechanical resistance against loads. On the other hand, RCM presented a significantly lower density (248.7 Kg/m^3^) associated with its constituent materials, including XPS and PUR, which are low-density materials (40.82 and 40.03 Kg/m^3^, respectively) due to the presence of interstitial voids [45]. Table 2 shows the densities of OTP and RCM.

**Figure 4:**
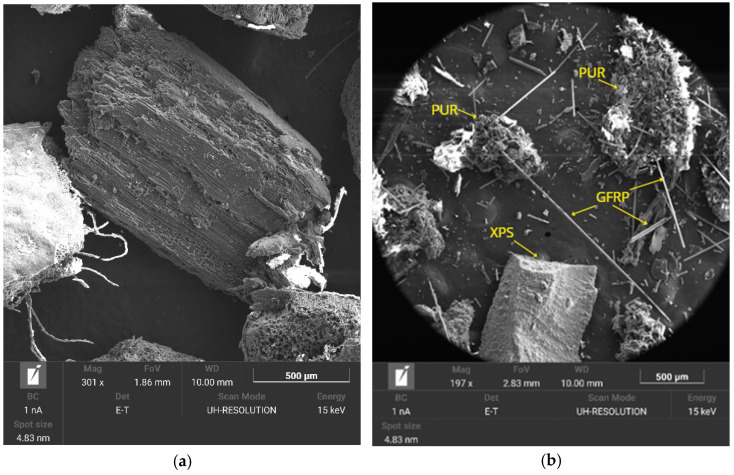
SEM of (**a**) OTP and (**b**) RCM.

The morphology of the parent materials is critical in the particleboard forming stage to facilitate particle adhesion and influences key aspects such as compaction, internal particle distribution and mechanical properties [24,46]. Figure 4 shows the SEM micrographs of (a) OTP and (b) RCM.

The SEM image of OTP (Figure 4a) showed a morphology with a rough surface where longitudinal fibers characteristic of this lignocellulosic residue were observed. This fibrous structure favors mechanical strength, as it allows better stress transfer through the matrix, especially in formulations with a higher proportion of OTP [47,48]. However, the same morphology may hinder the homogeneous compaction of the particleboard during the pressing process due to the tendency of the fibers to align and generate voids between particles. RCM showed an irregular morphology due to the combination of its components (Figure 4b). The XPS present in RCM exhibited more regular morphology and pores with smooth surfaces associated with the machining process. This fact may limit the interaction with the adhesive. PUR showed a more porous morphology. However, this morphology is conducive to low bulk density associated with porosity and the presence of interstitial voids and entrapped air in their structure [49]. In addition, elongated fibers corresponding to GFRP were observed.

**Figure 5:**
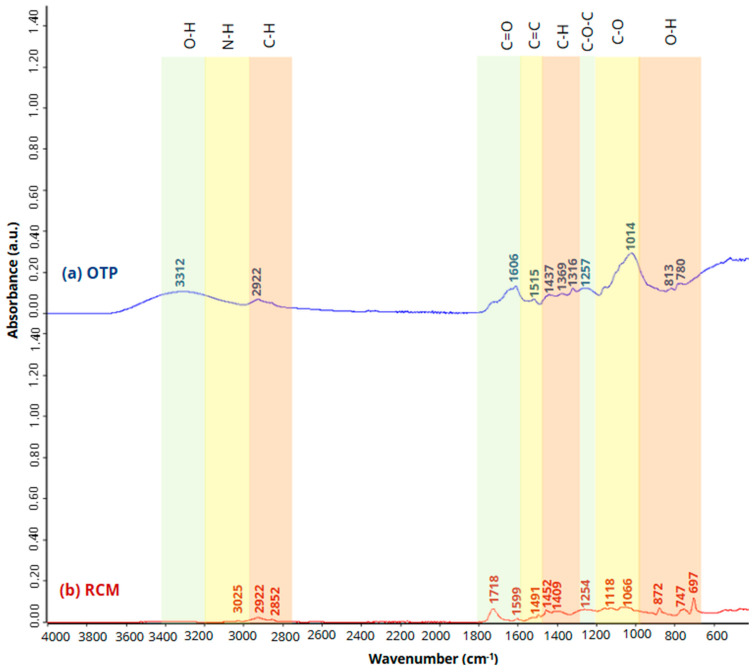
FTIR patterns of (**a**) OTP and (**b**) RCM.

The identification of the functional groups was performed on Bruker’s Vertex 70 FT-IR equipment (Bruker AXS GmbH, Karlsruhe, Germany) by Fourier transform infrared spectroscopy (FTIR) in a spectral range of 4000 to 400 cm^−1^ with a standard spectral resolution of 4 cm^−1^. Figure 5 shows the OTP and RCM spectra.

The band observed at 3312 cm^−1^ corresponds to the asymmetric O-H stretching vibration [47,50,51], while the band at 3025 cm^−1^ is associated with the asymmetric N-H stretching vibration characteristic of RCM urethanes [51,52,53]. The peaks observed at 2922 and 2852 cm^−1^ are associated with the asymmetric C-H stretching vibration characteristic of organic compounds [51,52]. The bands at 1599, 1606 and 1718 cm^−1^ are associated with the C=O asymmetric stretching vibration of urethanes [15,53,54,55]. The bands at 1500 cm^−1^ (1515 and 1491 cm^−1^) are associated with the C=C stretching vibration typical of aromatic rings [56,57], while those appearing between 1452 and 1316 cm^−1^ correspond to the deformation of the C-H bond [15,47] characteristic in cellulose-containing materials. On the other hand, bands appeared at 1257 and 1254 cm^−1^ associated with the vibration of the C-O-C bond [58]. The peaks observed between 1118 and 1014 cm^−1^, corresponding to C-O stretching vibration, are associated with the presence of cellulose and hemicellulose in OTP and RCM [50,51,59]. Finally, between 827 and 697 cm^−1^, O-H bending corresponding to the aromatic compounds present are observed [60]. Table 3 shows the characteristic peaks of the FTIR performed on OTP and RCM.

Differential thermogravimetric analysis (TG-DSC) was performed using the Metler Toledo analyzer (Metler Toledo, S.A., Barcelona, Spain) to evaluate the thermal stability of the materials that make up the particleboard. The test conditions were established by heating from ambient temperature up to 500 °C in air atmosphere at a heating rate of 10 °C/min.

The thermal degradation of OTP in air atmosphere showed 3 phases with a total mass loss of 65%. The first mass loss between 35 and 122 °C was 8% and was associated with the removal of water contained in OTP [15]. In the second phase, a weight loss of 28% occurred between 177 and 293 °C, associated with the decomposition of cellulose, hemicellulose and lignin [61]. The endothermic peak located at 272 °C is associated with the thermal degradation of these lignocellulosic components. Finally, in the third phase (between 331 and 500 °C), a total mass loss of 65% associated with the decomposition of residual cellulose and hemicellulose was presented [62].

The thermal stability of the RCM showed that the thermal decomposition of the material also occurs in 3 phases. In the first phase (between 75 and 117 °C), the mass loss was 3% and was associated with the breakdown of the polymeric chains of the RCM constituent materials, with the evaporation of the smaller molecules and with the decomposition of the urethane groups corresponding to the PUR [63]. The second phase, produced between 181 and 299 °C, showed a mass loss of 17%, while in the final phase, the mass loss slowed down and the total was 52%.

**Figure 6:**
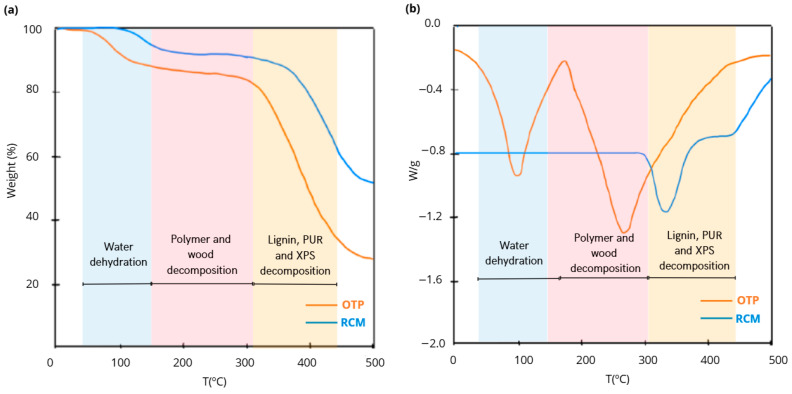
(**a**) TGA and (**b**) DTG patterns of OTP and RCM.

Figure 6 shows the TG and DSC curves of OTP and RCM performed in air atmosphere up to 500 °C. Table 4 provides the parameters obtained from the test results.

The thermal conductivity of OTP and RCM was determined on the Netsch HFM 446 Lambda Eco-Line (NETZSCH-Gerätebau GmbH, Selb, Germany). The values obtained are shown in Table 5.

### 2.2. Methodology and Characterization

Five series of mixtures with different OTP/RCM ratios (100:0, 70:30, 50:50, 30:70 and 0:100) were developed to form particleboards of dimensions 30 × 30 × 1.5 cm. Neopur 1791 PUR resin (26.25%) together with a corresponding hardener (8.75%) was used as a binder. According to the manufacturer’s safety data sheet, the PUR resin used (Neopur 1791) is not classified as flammable according to current regulations, which indicates that it does not present a significant risk of ignition under normal conditions of use. This feature represents an advantage in terms of fire safety for potential applications in construction. The weight ratio of particles to resin was 60:40. The solid/liquid (S/L) ratio remained constant (1.9) in all 5 series of panels. Table 6 shows the ratios of each of the formulations developed.

**Figure 7:**
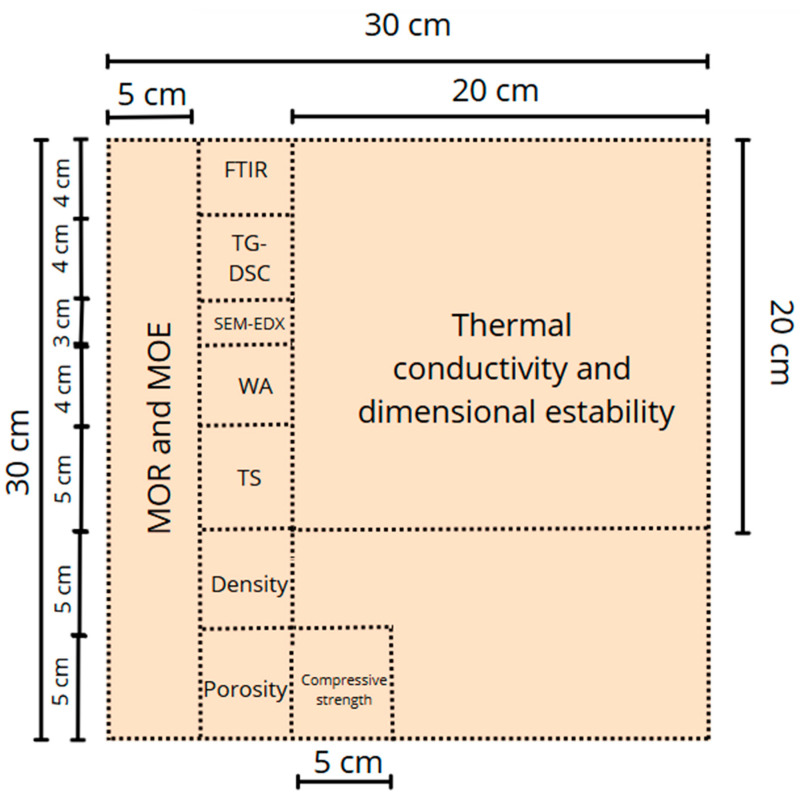
Particleboard cutting diagram.

The binder material was prepared in a Proeti planetary mixer (Proetisa S.A., Madrid, Spain) by mixing the resin (26.25%) and the corresponding hardener (8.75%) until a homogeneous solution was obtained. The technical workability parameters of this PUR resin, including temperature and open time, are specified in Table 1, according to data provided by the manufacturer. On the other hand, the solids were mixed manually in the proportions defined in Table 6 and then integrated with the binder. The resulting mixture was poured into a mold of dimensions 300 × 300 × 15 mm and subjected to a pressure of 15 MPa in a Shimadzu AG-300 KNX press (Shimadzu, Korneuburg, Austria) without application of temperature, maintaining the load for 20 h at a controlled temperature of 20 ± 2 °C. After this period of time, the particleboards were demolded to be subjected to the experimental phase. Three particleboards were manufactured from each of the formulations. Each of the boards was cut into various sizes for each of the tests according to Figure 7. In addition, three 50 × 50 × 50 mm specimens of each formulation were manufactured.

**Figure 8:**
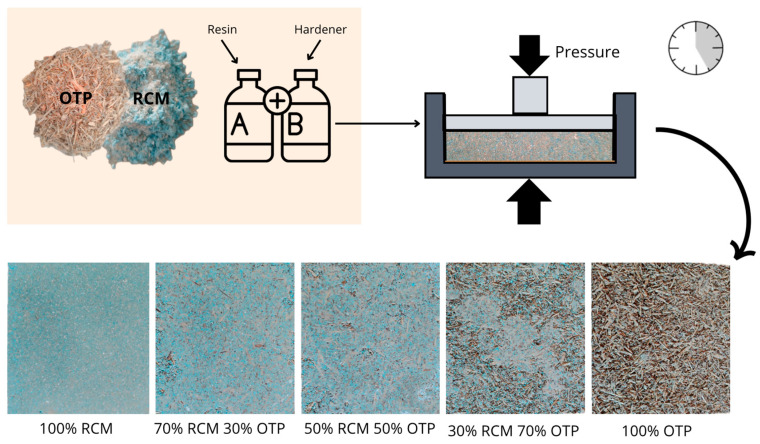
Particleboard manufacturing process and samples (A: resin, B: hardening agent).

Figure 8 shows schematically the methodology used in the preparation and shaping of the particleboards.

All particleboards developed were tested under the standards, equipment and methodologies shown in Table 7.

The test plan was organized in 5 phases: physical, chemical, thermal, structural and microscopic characterization. The first phase consisted of the physical characterization of the particleboards by means of dimensional stability (UNE-EN 1604:2013) [64], density (UNE-EN 323:1994) [65], porosity, water absorption (WA), thickness swelling (UNE-EN 317:1994) [66] and the determination of the contact angle (WCA) tests. In the second phase of chemical analysis, FTIR analysis was performed. The third phase integrated thermal characterization with thermal conductivity (UNE-EN 12667:2002) [67] and TG-DSC tests. The mechanical characterization included the three-point bending test for the determination of modulus of rupture (MOR) and modulus of elasticity (MOE) (UNE-EN 310:1994) [68] and internal bond strength (IB) (UNE-EN 319:1994) [69]. In addition, compressive strength was determined (UNE-EN 826:2013 [70]). Finally, the last phase consisted of microscopic characterization by microstructural analysis from SEM.

#### 2.2.1. Dimensional Stability

To evaluate the dimensional stability of particleboards under variable humidity conditions, controlled tests were carried out in a Dycometal SSC 140 climatic chamber (Dycometal, S.L., Barcelona, Spain). Three specimens per formulation, with dimensions of 200 × 200 mm, were arranged vertically inside the chamber, ensuring uniform air circulation around each specimen.

The test protocol consisted of subjecting the specimens to a relative humidity of 30 ± 5% and a temperature of 23 ± 2 °C for a period of 24 h. Subsequently, the relative humidity was increased to 50 ± 5% for an additional 3 h, keeping the temperature constant. The dimensional variation of the specimens before and after the test was measured using a digital caliper.

#### 2.2.2. Density and Porosity

Bulk density and porosity are intrinsically associated parameters in particleboards since the volume fraction occupied by pores directly influences the mass per unit volume. A higher pore index translates into a lower density, which significantly affects the strength-to-weight ratio of the material. This ratio, in turn, conditions fundamental mechanical properties such as flexural or compressive strength [71].

For physical characterization, three particleboards of each formulation with dimensions of 50 × 50 mm were tested. Density was determined by the ratio of mass to volume of each sample, calculated from their physical dimensions measured with precision digital calipers.

Internal porosity was evaluated by computed microtomography analysis using Bruker SkyScan 2214 equipment (Bruker AXS GmbH, Karlsruhe, Germany). Scan resolution was set at a voxel size of 6.1 μm. The analysis included an exposure time of 1.8 s per projection, with a rotation angular step of 0.4° and a total of 897 projections, which allowed the pore volume to be quantified nondestructively.

#### 2.2.3. Water Absorption (WA) and Thickness Swelling (TS)

The hygroscopic behavior of the particleboards was evaluated by determining the water absorption (WA) by immersion and the thickness swelling (TS) to determine the water stability. The water absorption of particleboards allows determining the maximum saturation degree reached by the material. This parameter is conditioned by the affinity of water with particleboards composed of biomass [72].

The experimental procedure consisted of vertically immersing 3 specimens of 50 × 50 mm of each of the formulations in water at a constant temperature of 20 ± 1 °C in a thermostatic bath. The test was performed at two times (2 and 24 h) to evaluate the initial water absorption of the particleboards and the response to prolonged exposure. WA was calculated by the mass difference quotient before and after immersing the samples in water, while TS was calculated from the dimensional variation of the thickness determined by means of a digital precision caliper.

#### 2.2.4. Thermal Conductivity

The thermal conductivity of the particleboards was analyzed in the Netsch HFM 446 Lambda Eco-Line equipment (Netzsch, High Franconia, Germany). Three 200 × 200 mm specimens of each of the formulations, which were previously weighed and measured, were analyzed for the test.

The equipment is composed of dual heat flow transducers placed between two isothermal plates to determine the temperature gradient through the thickness of the specimens.

Thermal conductivity values (λ) were calculated from the stabilized heat flux and the thermal gradient between the two surfaces, which allowed evaluating the insulating capacity of the particleboards.

#### 2.2.5. TGA-DSC

The thermal stability of the particleboards was analyzed by differential thermogravimetric analysis (TGA-DSC) on the Metler Toledo analyzer (Metler Toledo, S.A., Barcelona, Spain). The test conditions were set at 900 °C in an air atmosphere at a heating rate of 10 °C/min. The samples were subjected to an air flow rate of 40 mL/min.

#### 2.2.6. Water Contact Angle (WCA)

The wettability of the particleboards was determined by measuring the contact angle (WCA) in the Krüs Easy Drop equipment (Krüss optronic Gmbh, Hamburg, Spain). A 50 × 50 mm specimen per formulation was used for the test. WCA was measured on the surface by depositing a 10 ± 1 µL drop of distilled water. The initial contact angle was measured at time 0 and the final angle after 60 s.

#### 2.2.7. Flexural Strength, Modulus of Rupture (MOR) and Modulus of Elasticity (MOE)

The mechanical strength of the particleboard was evaluated by means of the three-point flexural test to determine both the maximum bending strength (MOR) and the modulus of elasticity in bending (MOE). This test allows analyzing the structural behavior of materials under transverse loads.

For this purpose, three specimens of dimensions 300 × 50 × 15 mm were prepared for each formulation. The test was carried out on a Zwick/Roell ProLine 10 kN universal testing machine (ZwickRoell S.L., Sant Cugat del Vallès, Spain) equipped with a two-point support device, a centered loading head and a 240 mm span, ensuring uniform load distribution over the upper surface of the specimen. The load application rate was 5 mm/min.

During the test, the maximum load supported prior to rupture was recorded, which allowed the MOR to be calculated. Deformations in the initial elastic range of the load–deformation curve were also recorded, from which the MOE was determined.

#### 2.2.8. Internal Bond Strength (IB)

The internal bond strength (IB) of particleboard was evaluated to determine the internal cohesion of the material, i.e., the ability of the bonded particles to hold together under a tensile load perpendicular to the plane of the board.

The test was performed using a Zwick/Roell ProLine 10kN universal tensile testing machine. Three specimens per formulation with standard dimensions of 50 × 50 × 50 mm were tested. The specimens were conditioned according to the UNE-EN 319:1994 standard [69]. Each specimen was bonded on its upper and lower faces to steel blocks using an epoxy adhesive. Once the adhesive had cured, the specimens were placed vertically in the tensile equipment and a force perpendicular to the plane of the board was applied until breakage was reached.

IB was calculated as the quotient between the maximum applied load and the cross-sectional area of the specimen. This value was used to estimate the quality of the bond between the particles and the binder used.

#### 2.2.9. Compressive Strength

The compressive strength of particleboard was evaluated in order to analyze the behavior of the material under load stresses applied perpendicular to the plane of the board.

The procedure was carried out by testing three specimens per formulation with dimensions of 50 × 50 mm using the Zwick/Roell ProLine 10 kN universal machine. The test was carried out at a constant displacement speed by placing the specimens between two plates.

## 3. Results

### 3.1. Physical Characterization

Table 8 shows the results obtained after the experimentation phase of the physical properties of the particleboards. The tests were carried out 24 h after the manufacturing and curing process at 20 ± 2 °C.

#### 3.1.1. Determination of Dimensional Stability

**Figure 9:**
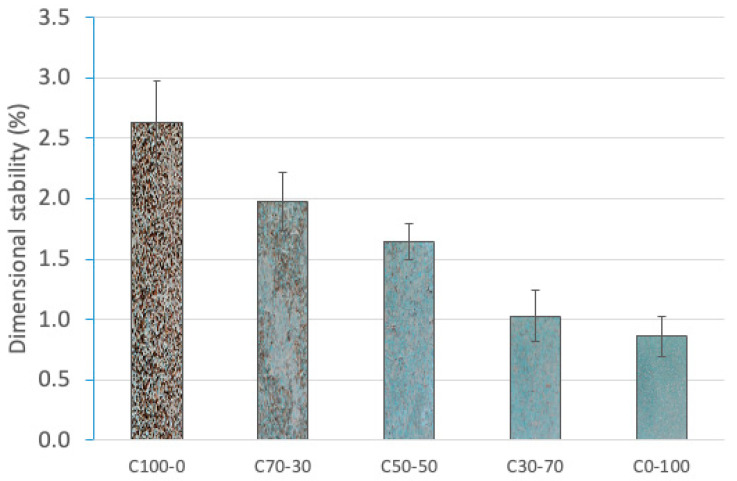
Dimensional stability (%) of particleboards.

The dimensional stability of the particleboard is directly influenced by the amount of moisture absorbed during testing, especially in composites with lignocellulosic components. Figure 9 shows the decreasing trend in dimensional variation as the OTP content in the board formulation decreases. This trend responds to the hygroscopic nature of OTP, whose composition, rich in cellulose and hemicellulose, makes it prone to absorb water under high-relative-humidity conditions, causing dimensional expansion and deformation phenomena [73].

On the contrary, formulation C0-100 presented the lowest dimensional variation recorded (0.86%), which is evidence of high stability against humidity. This behavior is related to the composition of the RCM that includes materials characterized by a low affinity for water and an internal structure with a lower moisture-retention capacity [74].

The intermediate formulations (C70-30, C50-50 and C30-70) showed progressively lower values of dimensional variation as the proportion of RCM increases, confirming the stabilizing effect of these materials on the board structure.

#### 3.1.2. Determination of Density and Porosity

**Figure 10:**
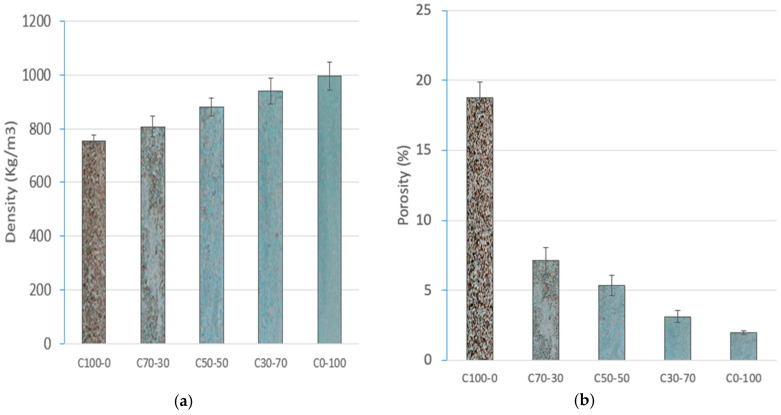
(**a**) Density (Kg/m^3^) and (**b**) porosity (%) of particleboards.

Density is directly related to the porosity and void volume present in the particleboards. Figure 10a shows the density values obtained where an upward trend is observed as the proportion of RCM in the particleboard increases despite the fact that the density of RCM is lower than that of OTP (Table 2). C100-0 presented the lowest density value (752.67 kg/m^3^), while C0-100 showed the highest density (995.61 kg/m^3^). This fact is justified by the compaction of the material during pressing. OTP, being composed mainly of lignocellulosic fibers, has a less compact structure, with a higher content of air or interstitial voids. On the contrary, RCM is constituted by particles derived from rigid foams (XPS and PUR) and GFRP fragments, resulting in a higher compaction under pressing and, therefore, higher density values.

Figure 10b verifies the inverse relationship between the density and porosity of the boards. As the amount of RCM in the formulation increases, the porosity decreases. C100-0 presents a porosity of 18.75%, while C0-100 reduces this value to 1.96%. This reduction is a direct consequence of the greater packing capacity of the polymeric materials, which allows for a denser internal configuration, with less volume of internal voids or voids.

#### 3.1.3. Determination of Water Absorption (WA) and Thickness Swelling (TS)

**Figure 11:**
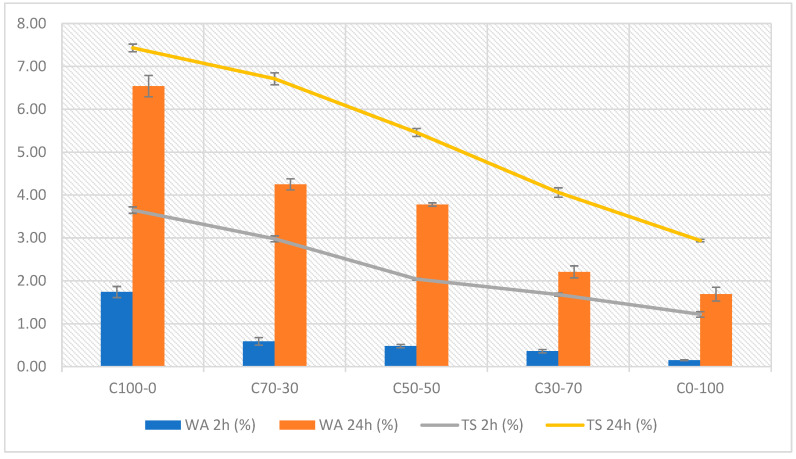
Water absorption (WA) and thickness swelling after immersion in water for 2 and 24 h of particleboards.

Figure 11 shows the results obtained in the particleboard hygroscopic behavior tests, evaluated through water absorption (WA) and thickness swelling (TS) after 2 and 24 h of immersion.

The results show a decreasing trend in both parameters as the RCM content in the formulation increased. C100-0 presented the highest values of water absorption (6.54%) and swelling (7.43%) after 24 h of immersion. This behavior is due to the hydrophilic nature and high porosity of lignocellulosic materials, whose fibrous structure favors capillarity and water retention [73].

C0-100 showed lower values of absorption (1.69%) and swelling (2.94%). This improvement is attributed to the lower affinity of the polymeric materials for water and to its denser and more closed structure, which limits permeability and moisture diffusion inside the material [62,74,75]. In addition, this formulation presented higher densification during the forming process, leading to a reduction in the void volume and, therefore, a lower absorption capacity.

#### 3.1.4. Water Contact Angle (WCA)

**Figure 12:**
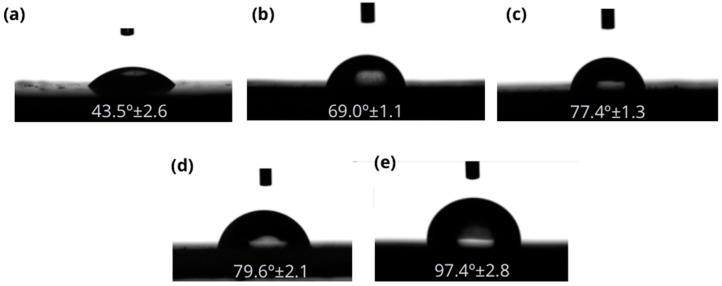
WCA (°) of (**a**) C100-0, (**b**) C70-30, (**c**) C50-50, (**d**) C30-70 and (**e**) C0-100.

The determination of the wettability is relevant to evaluate the adhesion capacity between the particles and the binder used. The WCA showed the wettability of particleboards where the lower this parameter, the higher the wettability. The replacement of RCM by OTP reduced free OH, present in cellulose, hemicellulose and lignin, which have an affinity for water, facilitating the formation of hydrogen bridges and, consequently, favoring moisture absorption [25]. On the other hand, RCM compositional materials present hydrophobic surfaces. Figure 12 shows that C100-0 presented the lowest WCA (43.5° ± 2.5) by producing a higher dispersion of the water droplet. On the contrary, C0-100 showed the highest WCA value (97.4° ± 2.8).

### 3.2. Chemical Characterization

#### FTIR

**Figure 13:**
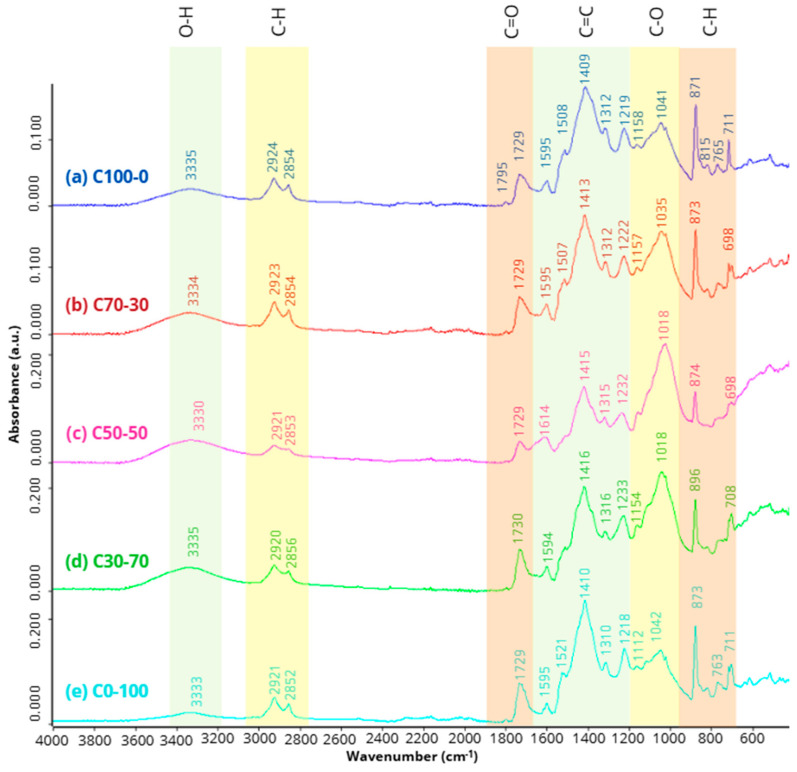
FTIR patterns of: (**a**) C100-0, (**b**) C70-30, (**c**) C50-50, (**d**) C70-30 and (**e**) C0-100.

Figure 13 shows the FTIR spectra for the five particleboard formulations. The wide bands in the region between 3335 and 3330 cm^−1^ are attributed to the stretching vibrations of the hydroxyl (O-H) group. This signal, of varying intensities between formulations, reflects the presence of hydrogen bonding due to residual moisture and polar functional groups present in the cellulose, hemicellulose and lignin of the OTP. Their width and displacement suggest different degrees of hydrophilic interactions between the PUR resin matrix and the particles, especially depending on the ratio of OTP and RCM.

In the 2924–2852 cm^−1^ range, bands characteristic of asymmetric C-H bond stretching are observed, associated with organic components of both OTP and RCM. These peaks confirm the presence of compounds derived from lignocellulosic materials and synthetic polymers [22,76,77,78]. The bands located in the region 1795–1729 cm^−1^ correspond to the stretching vibrations of the carbonyl group (C=O), indicative of the formation of urethane groups derived from the reaction between the isocyanate groups (-NCO) of the PUR resin and the hydroxyl groups of the OTP [15,22,78].

In contrast, the low surface reactivity of the components present in the RCM (XPS, recycled PUR and GFRP) may limit the formation of chemical bonds, so their contribution to the carbonyl signal is mainly interpreted as part of the unreacted polymer matrix. In the regions 1614–1218 cm^−1^ and 1158–1018 cm^−1^, the observed bands correspond, respectively, to the stretching vibrations of the C=C (in aromatic rings) and C-O bonds of the resin structural backbone, confirming the integration of aromatic and aliphatic phases. Finally, peaks between 896 and 698 cm^−1^ are associated with C-H bond vibrations in aromatic rings, suggesting the presence of structural residues of recycled polymers contained in the RCM, as well as by-products of PUR resin curing [79].

Table 9 shows the characteristic peaks of the 5 particleboard formulations.

### 3.3. Thermal Characterization

#### 3.3.1. TGA-DSC

**Figure 14:**
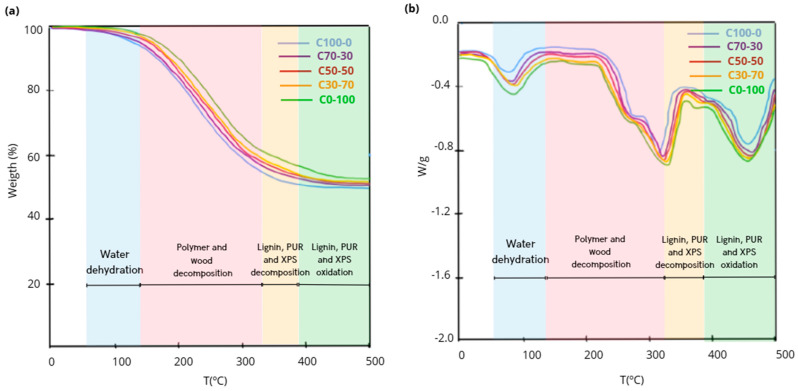
(**a**) TGA and (**b**) DTG patterns of particleboards C100-0, C70-30, C50-50, C30-70 and C0-100.

The thermogravimetric analysis performed showed the thermal stability of the formed particleboards. Differences were observed in the thermal degradation onset temperature of the five formulations. The thermal degradation onset temperature was lower in C100-0 (213 °C) due to the OTP content and the lower thermal stability of this material since hemicellulose decomposes at temperatures between 200 and 260 °C [15]. On the contrary, C0-100 presented the highest degradation temperature (383 °C) due to the content of materials with high thermal stability (Figure 14).

The results indicated that an 8% mass loss occurred at C100-0 between 68 and 129 °C. The loss is associated with the elimination of water absorbed by the OTP. In the second interval (between 130 and 306 °C), the mass loss was higher (47%) and is associated with the degradation of cellulose, hemicellulose and xylose from the OTP. In the third interval, a slower mass loss (up to 51%) was observed between 307 and 393 °C related to the loss of lignin [15,75]. In the last interval, between 394 and 500 °C, the sample suffered a slight mass loss (up to 54%) related to the final oxidation of the carbonaceous residues of the lignin. The thermogravimetric analysis of C0-100 showed a higher degradation temperature. The first range presented a mass loss of 6% between 76 and 135 °C related to the loss of water and solvents, while in the second range, between 136 and 309 °C, the mass loss was 41% as the RCM polymers decomposed. The total mass loss at C0-100 was 48% and is associated with the degradation of the polymeric materials.

These differences in the thermal degradation profiles have important practical implications. Formulations with a higher proportion of RCM exhibit higher temperature stability, which could be favorable for applications with moderate thermal requirements or where higher early-stage fire resistance is demanded, whereas OTP-rich formulations show a higher susceptibility to thermal degradation in the 200–400 °C range, which could limit their use in thermally hazardous environments if retardant additives are not incorporated.

Table 10 shows the temperature ranges and mass loss of the five formulations.

#### 3.3.2. Thermal Conductivity

**Figure 15:**
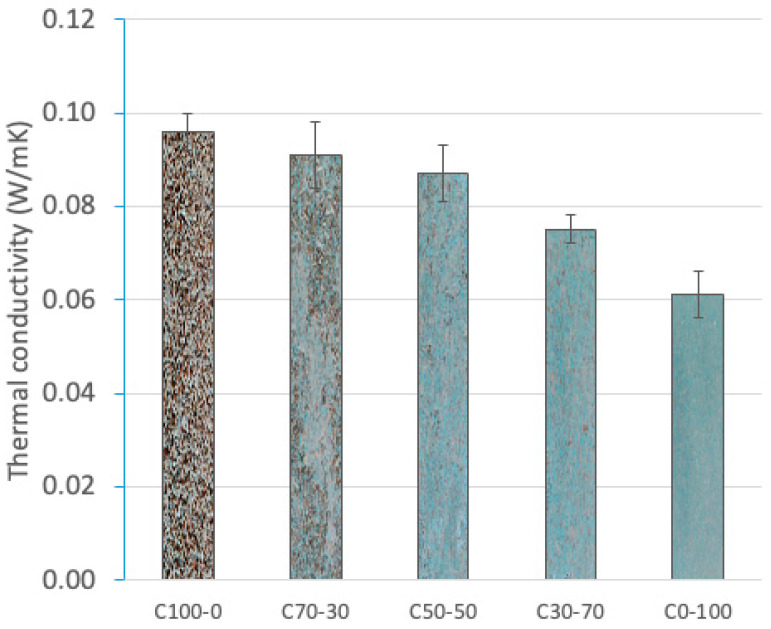
Thermal conductivity (W/mK) of particleboards.

The thermal conductivity of particleboards depends on the particleboard formulation. Figure 15 shows that the thermal conductivity values decrease as the RCM content in the particleboard increases. This fact is related to the thermal stability of the RCM constituent materials (PUR (0.024 W/mK) and XPS (0.032 W/mK)).

C100-0 presented the highest thermal conductivity (0.096 W/mK). This higher value can be attributed to the porosity of the lignocellulosic materials that favors the passage of heat through the board [15]. C0-100 showed the lowest thermal conductivity (0.061 W/mK). This result is related to the low thermal conductivity of the polymeric materials present in the RCM, as well as to the dispersed action of the glass fiber that contributes to breaking the thermal conduction paths [80,81]. On the other hand, the lower conductivity is associated with the denser structure.

The intermediate formulations (C70-30, C50-50 and C30-70) showed decreasing thermal conductivity values as the RCM content increased. This progressive trend confirms the role of polymeric residues in improving the thermal insulation of particleboards. Importantly, PUR resin contributes to improve the internal cohesion and compaction of particleboards, which can reduce the total porosity and, consequently, slightly decrease the thermal conductivity, especially in combinations with a higher RCM content.

### 3.4. Mechanical Characterization

Table 11 presents the results obtained in the mechanical strength tests of the particleboard.

The MOR values showed an increasing trend with an increasing RCM content in the mixture. The highest MOR values were obtained for C0-100 (7.11 MPa) and C100-0 (6.56 MPa), while the lowest value was obtained in C70-30 (5.40 MPa). This behavior is attributed to the GFRP contained in the RCM, which acts as reinforcement and provides higher mechanical strength. However, the value of C100-0 is justified by the good adhesion of the OTP with the PUR resin.

A progressive increase in MOE values was observed with increasing RCM. C0-100 showed the highest value (630 MPa). This evolution is related to the higher stiffness of the materials composing the RCM versus the lignocellulosic ones, which tend to show higher deformability under load. In addition, the presence of glass fibers and the lower porosity of RCM boards contribute to improve the elastic response of the material.

The IB parameter is reduced by 26.9% between C0-100 and C100-0, which is attributed to the porosity of C100-0 (18.75%) and the better adhesion between OTP particles. On the other hand, C0-100 having a denser structure with a lower porosity (1.96%) facilitated adhesive integrity. These factors also influence the compressive strength of the particleboards, obtaining values between 1.75 and 3 MPa.

### 3.5. Microscopic Characterization

#### SEM

**Figure 16:**
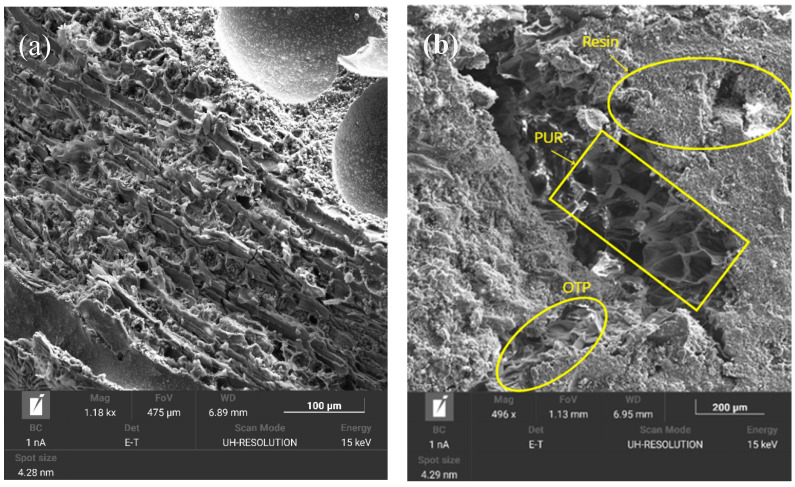

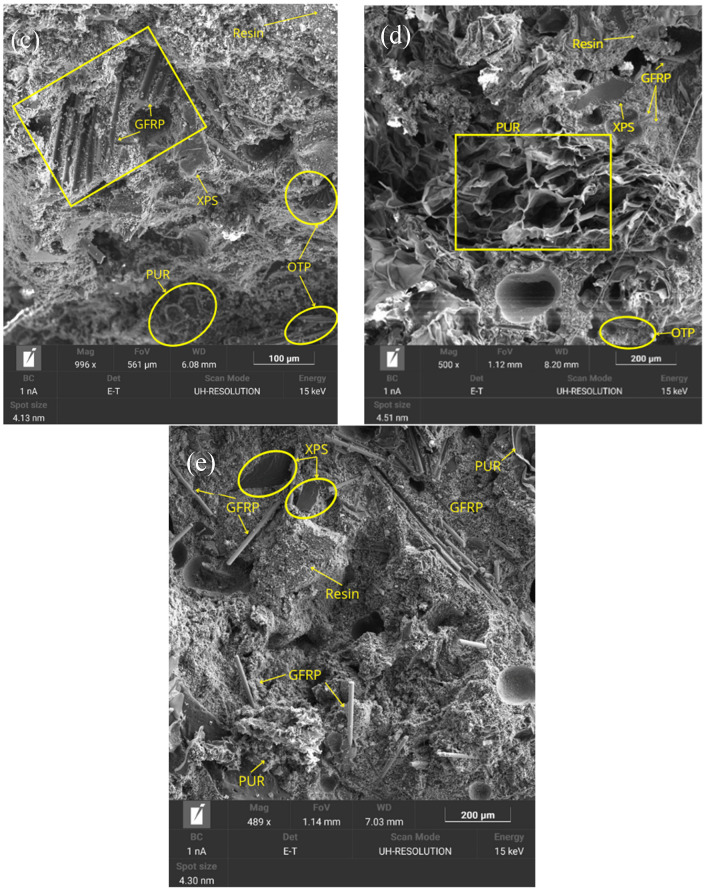
SEM secondary of (**a**) C100-0, (**b**) C70-30, (**c**) C50-50, (**d**) C30-70 and (**e**) C0-100.

The five particleboard formulations were analyzed by SEM with secondary electrons, in order to observe the surface microstructure and evaluate the quality of the particle distribution and interaction with the PUR resin. Although no quantitative analysis of the porosity or interface area was performed by image processing, this qualitative morphological study allowed the identification of relevant differences in the internal structure associated with the composition and density of the formulations. The results showed that the density and composition of the formulations influence the structural characteristics of the matrices. C0-100 (Figure 16e), with the highest density of 995.61 Kg/cm^3^, presented a more compact microstructure by decreasing the volume of voids due to the morphology of the constituent material. Therefore, it is corroborated that densification contributed to improve the mechanical properties by improving the stress transfer through the material. On the contrary, C100-0 (Figure 16a), with the lowest density of 752.67 Kg/cm^3^, showed a microstructure where spaces are observed between the OTP fibers. This arrangement is associated with the fibrous and less uniform morphology of the lignocellulosic material, which hinders compact packing and limits the effective contact between particles. The microstructure of the intermediate formulations (C70-30, C50-50 and C30-70) (Figure 16b–d) showed cohesive and compact matrices with a low void volume.

The SEM analysis also highlights that the PUR resin completely enveloped the surface of the RCM and OTP, ensuring the effective bonding of the materials forming the particleboard, as confirmed by FTIR analysis with peaks related to the presence of PUR resin. However, SEM images of C100-0 (Figure 16a) are evidence of the presence of interstitial voids associated with the fibrous and less uniform morphology of the lignocellulosic material of the OTP particles that hinder the compaction of the material and limit the effective contact between the particles.

In formulations with a high proportion of OTP, good wetting and adhesion between the fibers and resin is observed, attributed to the presence of hydroxyl groups (-OH) capable of reacting with the isocyanate groups (-NCO) of the PUR, forming covalent urethane bonds. On the other hand, the RCM-derived particles, with smoother and more chemically inert surfaces, show areas with less interfacial anchoring, suggesting a predominantly physical or mechanical adhesion. This difference in the chemical compatibility between the phases may partly explain the observed variability in the mechanical properties and moisture absorption behavior of the boards.

## 4. Conclusions

This study carried out a synthesis and development of particleboards made from olive grove and car industry by-products with five different formulations and studied their physical, chemical, mechanical, thermal and microstructural properties. From the results obtained, the influence of the particleboard composition was evaluated.

▪ The combination of lignocellulosic and synthetic components in adequate proportions allows the modulation of the hygroscopic behavior of particleboards, offering a balance between mechanical and structural properties and adapting to the needs of the material according to its final application.
▪ The particleboard with the highest proportion of RCM (C0-100) showed a thermal conductivity value of 0.061 W/mK, which complies with UNE-EN 13171:2013+A1:2015 [82] for thermal insulation products for building applications.
▪ The density of the particleboard influenced the porosity and thus the water absorption and swelling. This trend is associated with the porosity of OTP and its hydrophilic nature.
▪ Mechanical properties improved with the addition of RCM from C70-30 to C0-100, improving the MOR (from 5.40 to 7.11 MPa) and MOE (from 520 to 630 MPa) thanks to the structural reinforcement provided by the GFRP contained in the RCM.
▪ The thermogravimetric analysis showed the higher thermal stability of the particleboards with a higher RCM content (C0-100 and C30-70) due to the higher thermal stability of the RCM.
▪ The SEM results showed that the fibrous and porous structure of OTP makes it an alternative material for particleboard production when used at a suitable particle size. The inclusion of RCM in this type of board leads to a compact material with good mechanical properties and lower porosity.
▪ From an environmental point of view, the integration of RCM and OTP allows for a significant reduction in the carbon footprint, with greenhouse gas emission reductions of up to 95%, thanks to waste revaluation and mechanical recycling.
▪ OTP-based composites show mechanical properties comparable to standard MDF boards, although slightly lower in flexural strength [83,84], so they may be suitable for non-structural applications. However, they have advantages in terms of cost, by using residual raw materials, and recyclability, by reducing the use of virgin wood and facilitating its reintegration into production cycles.
▪ Although composites developed with OTP have shown a good mechanical performance and dimensional stability against moisture, this study has relevant limitations. Properties such as aging resistance, long-term durability and behavior under extreme hygrothermal conditions have not been evaluated. These aspects should be addressed in future research to validate their feasibility in structural or outdoor applications.
▪ The proposed manufacturing process is technologically feasible and scalable, being based on conventional operations (crushing, mixing and pressing) compatible with existing industrial lines, which facilitates its implementation without significant technical barriers.

Based on these conclusions, the valorization of OTP and RCM can reduce the dependence on wood as a raw material in the manufacture of particleboards, mitigating deforestation and contributing to the reduction of climate change.

## Figures and Tables

**Table 1:** Technical parameters resin Neopur 1791.

Resin Type	Curing Temperature (°C)	Time Types	Waiting Times (Minutes)	Viscosity (cps)	Density (Kg/m^3^)
Neopur 1791	20–22 °C	Open time	40–45	200–250 (25 °C)	1.620
		Hardening time	300–480		
		Curing time	1440		

**Table 2:** Density of OTP and RCM.

Raw Materials	Density (Kg/m^3^)
OTP	427.7 ± 2.05
RCM	248.7 ± 1.52

**Table 3:** Characteristic absorption peaks of FTIR of OTP and RCM.

Function Group	Wavenumber Range (cm^−1^)	FTIR Peaks (cm^−1^)		References
Raw Materials		OTP	RCM	
Stretching vibration O-H	3312	3312	-	[47,50,51]
Stretching vibration N-H	3025	-	3025	[51,52,53]
Stretching vibration C-H	2922–2852	2922	2922, 2852	[51,52]
Asymmetric stretching vibration C=O	1718–1599	1606	1718, 1599	[15,53,54,55]
Stretching vibration C=C	1515–1491	1515	1491	[56,57]
C-H deformation	1452–1316	1437–1316	1452, 1409	[15,47]
Stretching C-O-C	1257–1254	1257	1254	[58]
Asymmetric stretching vibration C-O	1118–1014	1014	1118, 1066	[50,51,59]
Bending O-H	872–697	813, 780	872–697	[60]

**Table 4:** Parameters obtained from TG-DSC results of thermal degradation of OTP and RCM.

Atmosphere	Raw Material	Temperature Range (°C)	Weight Loss (%)
Air	OTP	35–122	8
		123–303	28
		304–500	65
	RCM	75–117	3
		118–375	17
		376–500	52

**Table 5:** Thermal conductivity (W/mK) of OTP and RCM.

Raw Material	Thermal Conductivity (W/mK)
OTP	0.091
RCM	0.027

**Table 6:** Mix proportions of particleboards.

Specimen	OTP (%w)	RCM (%w)	Resin (%w)	Hardening Agent (%w)	S/L
C100-0	65.00	0.00	26.25	8.75	1.9
C70-30	45.50	19.50	26.25	8.75	1.9
C50-50	32.50	32.50	26.25	8.75	1.9
C30-70	19.50	45.50	26.25	8.75	1.9
C0-100	0.00	65.00	26.25	8.75	1.9

**Table 7:** Parameter, standard and equipment used.

Parameter	Standard	Equipment
Dimensional stability	UNE-EN 1604:2013 [64]	Moisture chamber Dycometal SSC 140 (Dycometal, S.L., Barcelona, Spain)
Density	UNE-EN 323:1994 [65]	Balance RB-30KG Cobos (Cobos, Barcelona, Spain)
Porosity	-	SkyScan 2214 Bruker (Bruker AXS GmbH, Karlsruhe, Germany)
Water absorption (WA) and thickness swelling (TS)	UNE-EN 317:1994 [66]	Digital gauge
Thermal conductivity	UNE-EN 12667:2002 [67]	HFM 446 Lambda Eco-Line Netzsch (Netzsch, High Franconia, Germany)
Flexural strength, modulus of rupture (MOR) and modulus of elasticity (MOE)	UNE-EN 310:1994 [68]	Zwick/Roell ProLine 10 kN (ZwickRoell S.L., Sant Cugat del Vallès, Spain)
Internal Bond Strength (IB)	UNE-EN 319:1994 [69]	Zwick/Roell ProLine 10 kN (ZwickRoell S.L., Sant Cugat del Vallès, Spain)
Compressive strength	UNE-EN 826:2013 [70]	Zwick/Roell ProLine 10 kN (ZwickRoell S.L., Sant Cugat del Vallès, Spain)
Water Contact Angle (WCA)	-	Krüss Easy Drop (Krüss optronic Gmbh, Hamburg, Spain)
XRD	-	X’Pert Pro PANalytical (Malvern Panalytical, Almelo, The Netherlands)
FTIR	-	FT-IR Vertex 70 Bruker (Bruker AXS GmbH, Karlsruhe, Germany)
TG-DSC	-	Metler Toledo (Metler Toledo, S.A., Barcelona, Spain)
SEM	-	Microscope Carl Zeiss Merlin (Carl Zeiss AG, Oberkochen, Germany)

**Table 8:** Results of physical parameters of particleboards.

Specimen	Dimensional Stability (%)	Density (Kg/m^3^)	Porosity (%)	WA 2 h (%)	WA 24 h (%)	TS 2 h (%)	TS 24 h (%)	WCA (°)
C100-0	2.63 ± 0.35	752.67 ± 24.3	18.75 ± 1.12	1.74 ± 0.13	6.54 ± 0,25	3.65 ± 0.07	7.43 ± 0.09	43.5 ± 2.6
C70-30	1.98 ± 0.24	806.52 ± 38.5	7.12 ± 0.97	0.59 ± 0.09	4.25 ± 0.13	2.98 ± 0.06	6.71 ± 0.14	69.0 ± 1.1
C50-50	1.65 ± 0.15	881.04 ± 33.1	5.36 ± 0.70	0.48 ± 0.04	3.78 ± 0.04	2.04 ± 0.02	5.46 ± 0.09	77.4 ± 1.3
C30-70	1.03 ± 0.21	939.87 ± 48.7	3.14 ± 0.42	0.36 ± 0.04	2.21 ± 0.14	1.68 ± 0.03	4.06 ± 0.11	79.6 ± 2.1
C0-100	0.86 ± 0.17	995.61 ± 51.9	1.96 ± 0.13	0.15 ± 0.01	1.69 ± 0.16	1.22 ± 0.06	2.94 ± 0.03	97.4 ± 2.8

**Table 9:** Characteristic absorption peaks of FTIR of particleboards.

Function Group	Wavenumber Range (cm^−1^)	FTIR Peaks (cm^−1^)					References
Raw Materials		C100-0	C70-30	C50-50	C30-70	C0-100	
Stretching vibration O-H	3335–3330	3335	3334	3330	3335	3333	[15,22,50,77]
Stretching vibration C-H	2924–2852	2924, 2854	2923, 2854	2921, 2853	2920, 2856	2921, 2852	[22,76,77,78]
Asymmetric stretching vibration C=O	1795–1729	1795, 1729	1729	1729	1730	1729	[15,22,78]
Stretching vibration C=C	1614–1218	1595–1219	1595–1232	1614–1232	1594–1233	1595–1218	[79]
Asymmetric stretching vibration C-O	1158–1018	1158, 1041	1157, 1035	1157, 1018	1154, 1018	1112, 1042	[59,76,78]
Stretching vibration C-H	896–698	871–711	873, 698	874, 698	896, 708	873–711	[79]

**Table 10:** Temperature range and mass loss of samples.

Atmosphere	Sample	Temperature Range (°C)	Weight Loss (%)
Air	C100-0	68–129	8
		130–306	47
		307–393	51
		394–500	54
	C70-30	69–129	7
		130–306	45
		307–393	49
		394–500	52
	C50-50	71–129	7
		130–308	44
		309–393	47
		394–500	52
	C30-70	73–132	6
		133–309	44
		310–393	48
		394–500	50
	C0-100	76–135	6
		136–309	41
		310–393	45
		394–500	48

**Table 11:** Results of mechanical parameters of particleboards.

Specimen	MOR (MPa)	MOE (MPa)	IB (MPa)	Compressive Strength (MPa)
C100-0	6.56 ± 0.08	612 ± 43	0.098 ± 0.008	3.05 ± 0.44
C70-30	5.40 ± 0.09	520 ± 51	0.110 ± 0.005	1.98 ± 0.13
C50-50	5.91 ± 0.05	560 ± 69	0.118 ± 0.006	1.75 ± 0.25
C30-70	6.32 ± 0.16	590 ± 40	0.126 ± 0.003	1.82 ± 0.09
C0-100	7.11 ± 0.12	630 ± 56	0.134 ± 0.004	2.94 ± 0.19

## Data Availability

The original contributions presented in this study are included in the article. Further inquiries can be directed to the corresponding author.

## Abbreviations

The following abbreviations are used in this manuscript:

OTP	Olive tree pruning
RCM	Recycled chopped material

## References

[1] Wang L., Chen S.S., Tsang D.C.W., Poon C.S., Shih K. (2016). Value-added recycling of construction waste wood into noise and thermal insulating cement-bonded particleboards. Constr. Build. Mater..

[2] Svobodová H., Hlaváčková P. (2023). Forest as a source of renewable material to reduce the environmental impact of buildings. J. For. Sci..

[3] Xu P., Tam V.W.Y., Li H., Zhu J., Xu X. (2025). A critical review of bamboo construction materials for sustainability. Renew. Sustain. Energy Rev..

[4] Ferreira Martins R.S., Gomes Gonçalves F., Costa Lelis R.C., Gutemberg Alcântara Segundinho P., Mota Nunes A., Baptista Vidaurre G., Silva Chaves I., Barros Santiago S. (2020). Physical properties and formaldehyde emission in particleboards of *Eucalyptus* sp. and ligno-cellulosic agroindustrial waste. Sci. For..

[5] Galán-Martín Á., Contreras M.d.M., Romero I., Ruiz E., Bueno-Rodríguez S., Eliche-Quesada D., Castro-Galiano E. (2022). The potential role of olive groves to deliver carbon dioxide removal in a carbon-neutral Europe: Opportunities and challenges. Renew. Sustain. Energy Rev..

[6] Fanourakis S., Romero-García J.M., Castro E., Jiménez-Esteller L., Galán-Martín Á. (2024). Economic and environmental implications of carbon capture in an olive pruning tree biomass biorefinery. J. Clean. Prod..

[7] Martín J.F.G., Cuevas M., Feng C., Mateos P.Á., García M.T., Sánchez S. (2020). Energetic Valorisation of Olive Biomass: Olive-Tree Pruning, Olive Stones and Pomaces. Processes.

[8] Catizzone E., Freda C., Villone A., Romanelli A., Cornacchia G. (2025). Gasification of olive tree pruning in a rotary kiln reactor integrated with radio frequency plasma torch. Fuel.

[9] Aguado R., Escámez A., Jurado F., Vera D. (2023). Experimental assessment of a pilot-scale gasification plant fueled with olive pomace pellets for combined power, heat and biochar production. Fuel.

[10] Soltero V.M., Román L., Peralta M.E., Chacartegui R. (2020). Sustainable biomass pellets using trunk wood from olive groves at the end of their life cycle. Energy Rep..

[11] Kougioumtzis M.A., Kanaveli I.P., Karampinis E., Grammelis P., Kakaras E. (2021). Combustion of olive tree pruning pellets versus sunflower husk pellets at industrial boiler. Monitoring of emissions and combustion efficiency. Renew. Energy.

[12] Nicodemou A., Kallis M., Koutinas M. (2023). Biorefinery development for the production of polyphenols, algal biomass and lipids using olive processing industry waste. Sustain. Chem. Pharm..

[13] Cubero-Cardoso J., Hernández-Escaño M., Trujillo-Reyes Á., Fermoso F.G., Fernández-Recamales M.Á., Fernández-Bolaños J., Rodríguez-Gutiérrez G., Urbano J. (2025). Mechanochemical-assisted Natural Deep Eutectic Solvent as a platform for an olive leaves biorefinery: Extraction of bioactive compounds and methane production. Sustain. Chem. Pharm..

[14] Oliva J.M., Negro M.J., Álvarez C., Manzanares P., Moreno A.D. (2020). Fermentation strategies for the efficient use of olive tree pruning biomass from a flexible biorefinery approach. Fuel.

[15] Ramos P.B., Jerez F., Erans M., Mamaní A., Ponce M.F., Sardella M.F., Sanz-Pérez E.S., Sanz R., Arencibia A., Bavio M.A. (2025). Environmentally valorization of olive tree pruning residue: Activated carbons for CO_2_ capture and energy storage in supercapacitors. Biomass Bioenergy.

[16] González-García S., Ferro F.S., Lopes Silva D.A., Feijoo G., Lahr F.A.R., Moreira M.T. (2019). Cross-country comparison on environmental impacts of particleboard production in Brazil and Spain. Resour. Conserv. Recycl..

[17] Tufan M.Z., Özel C., Üner B. (2025). Investigation of the effect of using multi-walled carbon nanotubes with various glues on particleboard properties using response surface methodology. Constr. Build. Mater..

[18] Souza A.G.O., Eufrade-Junior H.d.J., Spadim E.R., Guerra S.P.S., Esperancini M.S.T. (2024). Exploring the technical and economic viability of lignocellulosic waste briquettes from the wood panel industry. Ind. Crops Prod..

[19] Gu K., Zhang X., Dong Z., Chen H., Xu M., Sun Z., Han S., Zhang J., Yu Y., Hou J. (2025). Deep learning-aided preparation and mechanism revaluation of waste wood lignocellulose-based flame-retardant composites. Int. J. Biol. Macromol..

[20] FAOSTAT. https://www.fao.org/faostat/en/#data/FO.

[21] Lee S.H., Lum W.C., Boon J.G., Kristak L., Antov P., Pedzik M., Rogozinski T., Taghiyari H.R., Lubis M.A.R., Fatriasari W. (2022). Particleboard from agricultural biomass and recycled wood waste: A review. J. Mater. Res. Technol..

[22] Santos J., Fernandes R.A., Ferreira N., Ferreira I., Vieira C., Magalhães F.D., Martins J.M., de Carvalho L.H. (2024). New particleboards for food-packaging from poplar peeling by-products using a circular economy approach. Int. J. Adhes. Adhes..

[23] Rana C.J.F., Quintos-Cortiguerra A.L., Dorado A.B., Jimenez J.P. (2025). Influence of lacquer sanding sealer treatment on the properties of bamboo waste particleboards for sustainable handicrafts. Adv. Bamboo Sci..

[24] Audibert E., Ducceschi L., Quintero A., Martel F., Paës G., Rémond C. (2025). Binderless particleboards from steam exploded woody biomass: Chemical and morphological properties relate to their mechanical and physical behavior. Ind. Crops Prod..

[25] Yang S., Li M., Wang Y., Liu X., Qing Y., Li X., Wu Y., Liu M., Zhang X. (2024). Industrial-scale manufacturing of particleboards using agricultural waste camellia oleifera shells. Constr. Build. Mater..

[26] Güler C., Sarikaya A., Sertkaya A.A., Canli E. (2024). Almond shell particle containing particleboard mechanical and physical properties. Constr. Build. Mater..

[27] Moutousidis D., Karidi K., Athanassiadou E., Stylianou E., Giannakis N., Koutinas A. (2023). Reinforcement of urea formaldehyde resins with pectins derived from orange peel residues for the production of wood-based panels. Sustain. Chem. Environ..

[28] Zhao L., Li W., Cheng Y., Zhao J., Tian D., Huang M., Shen F. (2024). Preparation and evaluation of lignin-phenol-formaldehyde resin as wood adhesive using unmodified lignin. Ind. Crops Prod..

[29] Dorieh A., Farajollah Pour M., Ghafari Movahed S., Pizzi A., Pouresmaeel Selakjani P., Valizadeh Kiamahalleh M., Hatefnia H., Shahavi M.H., Aghaei R. (2022). A review of recent progress in melamine-formaldehyde resin based nanocomposites as coating materials. Prog. Org. Coat..

[30] de Paiva E.M., Mattos A.L.A., da Silva G.S., Canuto K.M., Leitão R.C., Alves J.L.F., de Brito E.S. (2024). Valorizing cashew nutshell residue for sustainable lignocellulosic panels using a bio-based phenolic resin as a circular economy solution. Ind. Crops Prod..

[31] Abobakr H., Kakou C.A., Bensalah M.O., Bouhfid R., Qaiss A.e.K., Raji M. (2025). Development of lightweight insulation particleboard with superior thermo-mechanical and acoustic properties based on rice husks. J. Build. Eng..

[32] Antoun K., Besserer A., el Hage R., Segovia C., Sonnier R., Brosse N. (2024). Environmentally-friendly, binder-free, non-flammable particleboard with enhanced properties. Ind. Crops Prod..

[33] Duran A.J.F.P., Campos Filho L.E., Lyra G.P., da Costa Held G.A., Rossignolo J.A., Fiorelli J. (2025). Evaluation of physical and mechanical properties in medium density particleboards made from sugarcane bagasse and plastic waste (HDPE) at different pressing temperatures. Ind. Crops Prod..

[34] Morandini M., Barbu M.C., Váňová R., Kain S., Tippner J., Petutschnigg A., Kristak L., Kain G., Sepperer T., Schnabel T. (2025). Valorization of Extracted Bark for Particleboard Production: A Life-Cycle Impact Assessment. Polymers.

[35] Regmi S., Bajwa D., Igathinathane C., Nahar N. (2022). High fiber fraction DDGS—A functional filler for manufacturing low-density particleboards. Ind. Crops Prod..

[36] Fehrmann J., Belleville B., Ozarska B., Ismayati M., Dwianto W. (2024). Effects of mat composition and pressing time on citric acid-bonded ultra-low-density hemp hurd particleboard. Ind. Crops Prod..

[37] Valenzuela Expósito J.J., Picazo Camilo E., Corpas Iglesias F.A. (2024). Development of a Modular Sandwich Panel with a Composite Core of Recycled Material for Application in Sustainable Building. Polymers.

[38] Proença M., Santos P., Godinho L., Neves e Sousa A., Correia J.R., Garrido M., Sena-Cruz J. (2024). Acoustic behaviour of GFRP-PUR web-core composite sandwich panels. Constr. Build. Mater..

[39] Benzo P.G., Pereira J.M., Sena-Cruz J. (2023). Structural response of steel sandwich panels with PUR foam core subjected to edgewise compression: Experimental, numerical, and analytical considerations. Constr. Build. Mater..

[40] Darzi S., Karampour H., Bailleres H., Gilbert B.P., Fernando D. (2020). Load bearing sandwich timber walls with plywood faces and bamboo core. Structures.

[41] Yan Z., Shen C., Fang H., Xie L., Bao X., Wang H. (2024). Compressive performance of full-scale GFRP composite sandwich wall panels with wood core. J. Build. Eng..

[42] Sohrabi M., Mohammadi H., León M., Armengol J. (2025). Detection of fungal trunk pathogens from wood tissues and pruning wood debris of olive trees in Iran. Physiol. Mol. Plant Pathol..

[43] Kougioumtzis M.A., Tsiantzi S., Athanassiadou E., Karampinis E., Grammelis P., Kakaras E. (2023). Valorisation of olive tree prunings for the production of particleboards. Evaluation of the particleboard properties at different substitution levels. Ind. Crops Prod..

[44] Aras U., Kalaycıoğlu H., Yel H., Kuştaş S. (2022). Utilization of olive mill solid waste in the manufacturing of cement-bonded particleboard. J. Build. Eng..

[45] Winkler-Skalna A., Łoboda B. (2020). Determination of the thermal insulation properties of cylindrical PUR foam products throughout the entire life cycle using accelerated aging procedures. J. Build. Eng..

[46] da Silva Cazella P.H., de Souza M.V., Rodrigues F.R., da Silva S.A.M., Bispo R.A., de Araujo V.A., Christoforo A.L. (2024). Polyethylene terephthalate (PET) as a recycled raw material for particleboards produced from pinus wood and biopolymer resin. J. Clean. Prod..

[47] Mamaní A., Sardella M.F., Giménez M., Deiana C. (2019). Highly microporous carbons from olive tree pruning: Optimization of chemical activation conditions. J. Environ. Chem. Eng..

[48] Mamaní A., Maturano Y., Mestre V., Montoro L., Gassa L., Deiana C., Sardella F. (2021). Valorization of olive tree pruning. Application for energy storage and biofuel production. Ind. Crops Prod..

[49] Oral N., Otuz O., Kocabıyık R.K., Akoğlu M. (2024). Characterization of mechanical and temperature effects on delamination and basic strength behaviors of PVC coated PUR foam substrate used in bus dashboard. Heliyon.

[50] Wang C., Zainal Abidin S., Toyong N.M.P., Zhu W., Zhang Y. (2024). Mildew resistance and antibacterial activity of plywood decorated with ZnO/TiO_2_ nanoparticle. J. Saudi Chem. Soc..

[51] Cao Y., Teng Y., Zhang P., Bao J., Feng P., Li R., Wang W. (2024). UV ageing of epoxy resin-based glass fiber-reinforced polymer composites incorporating with various curing agents. Mater. Today Commun..

[52] Ou Y., Sun Y., Guo X., Jiao Q. (2018). Investigation on the thermal decomposition of hydroxyl terminated polyether based polyurethanes with inert and energetic plasticizers by DSC-TG-MS-FTIR. J. Anal. Appl. Pyrolysis.

[53] Zhao X., Liu Y., Lv Y., Liu M. (2024). Research on lignin-modified flexible polyurethane foam and its application in sound absorption. J. Ind. Eng. Chem..

[54] Li S., Han K., Li J., Li M., Lu C. (2017). Preparation and characterization of super activated carbon produced from gulfweed by KOH activation. Microporous Mesoporous Mater..

[55] Jofrishal J., Adlim M., Yusibani E., Akhyar A., Rahmayani R.F.I., Fajri R. (2023). Preparation and characterization of indoor heat blockage panel composites made of polyurethane-hybrid-foam-concrete and rice-husk-ash. Heliyon.

[56] Lu J., Sun X., Chen Z., Jiang P., Li L., Wang M. (2024). Sandwich-structure inspired super-tough and fire-resistant plywood containing vinyl acetate-ethylene based adhesive reinforced by melamine amino trimethyl phosphate and sodium lignosulfonate. Polym. Degrad. Stab..

[57] Wang W., Sun S., Shi Y., Tang W., Cui S., Sun D., Liu H., Wang H., Jin X. (2024). The chemical bonding of extruded polystyrene foam with mortar through UV curable organic-inorganic hybrid coatings. Constr. Build. Mater..

[58] Santos J.I., Martín-Sampedro R., Fillat Ú., Oliva J.M., Negro M.J., Ballesteros M., Eugenio M.E., Ibarra D. (2015). Evaluating lignin-rich residues from biochemical ethanol production of wheat straw and olive tree pruning by FTIR and 2D-NMR. Int. J. Polym. Sci..

[59] Bekhta P., Sedliačik J., Kusniak I., Gryc V., Pipíška T., Ráheľ J., Lepcio P., Pavliňák D., Tymyk D., Chernetskyi O. (2024). Enhancing the properties of thermoplastic-bonded plywood by treating the birch veneers with citric acid. Int. J. Adhes. Adhes..

[60] Yorgun S., Yildiz D. (2015). Slow pyrolysis of paulownia wood: Effects of pyrolysis parameters on product yields and bio-oil characterization. J. Anal. Appl. Pyrolysis.

[61] Rico J.J., Pérez-Orozco R., Patiño Vilas D., Porteiro J. (2022). TG/DSC and kinetic parametrization of the combustion of agricultural and forestry residues. Biomass Bioenergy.

[62] Alrawashdeh K.A., Slopiecka K., Alshorman A.A., Bartocci P., Fantozzi F. (2017). Pyrolytic degradation of olive waste residue (OWR) by TGA: Thermal decomposition behavior and kinetic study. J. Energy Power.

[63] Jiao L., Xiao H., Wang Q., Sun J. (2013). Thermal degradation characteristics of rigid polyurethane foam and the volatile products analysis with TG-FTIR-MS. Polym. Degrad. Stab..

[64] (2013). Thermal Insulating Products for Building Applications–Determination of Dimensional Stability Under Specified Temperature and Humidity Conditions.

[65] (1994). Wood-Based Panels–Determination of Density.

[66] (1994). Particleboards and Fibreboards–Determination of Swelling in Thickness After Immersion in Water.

[67] (2002). Thermal Performance of Building Materials and Products.

[68] (1994). Wood-Based Panels–Determination of Modulus of Elasticity in Bending and of Bending Strength.

[69] (1994). Particleboards and Fibreboards–Determination of Tensile Strength Perpendicular to the Plane of the Board.

[70] (2013). Thermal Insulating Products for Building Applications–Determination of Compression Behaviour.

[71] Bekhta P., Kozak R., Gryc V., Pipíška T., Sedliačik J., Reh R., Ráheľ J., Rousek R. (2023). Properties of lightweight particleboard made with sunflower stalk particles in the core layer. Ind. Crops Prod..

[72] Santos M.M., Diez M.A., Suárez M., Centeno T.A. (2021). Innovative particleboard material from the organic fraction of municipal solid waste. J. Build. Eng..

[73] Oya-Monzón M., Eliche-Quesada D., La Rubia M.D. (2024). Effect of the Incorporation of Olive Tree Pruning Sawdust in the Production of Lightweight Mortars. J. Compos. Sci..

[74] Shi L., Hu C., Zhang W., Chen R., Ye Y., Fan Z., Lin X. (2024). Effects of adhesive residues in wood particles on the properties of particleboard. Ind. Crops Prod..

[75] Si S., Zheng X., Zhou C., Zou D., Li X. (2024). Enhancing the water resistance properties of bamboo particleboard by reconstructing lignocellulose through carbonization treatment. Int. J. Biol. Macromol..

[76] Zhao W., Yan W., Zhang Z., Gao H., Zeng Q., Du G., Fan M. (2022). Development and performance evaluation of wood-pulp/glass fibre hybrid composites as core materials for vacuum insulation panels. J. Clean. Prod..

[77] Betené A.D.O., Ndiwe B., Krishnan G.S., Wedaïna A.G., Tchoupmene C.M., Djakou C.B.N., Taoga M.M., Betené F.E., Atangana A. (2023). Processing of tropical agro-industrial waste for particleboard manufacture: Dimensional stability and mechanical performance. J. Build. Eng..

[78] Huang S., Yan L., Bachtiar E.V., Kasal B. (2022). Durability of epoxy and polyurethane bonded timber-hybrid FRP joints under hygrothermal and weathering conditions. J. Build. Eng..

[79] Huang S., Bachtiar E.V., Yan L., Kasal B. (2022). Bond behaviour and thermal stability of flax/glass hybrid fibre reinforced polymer–timber structures connected by polyurethane. Constr. Build. Mater..

[80] Faria D.L., Gonçalves F.G., Maffioletti F.D., Scatolino M.V., Soriano J., de Paula Protásio T., Lopez Y.M., Paes J.B., Mendes L.M., Junior J.B.G. (2024). Particleboards based on agricultural and agroforestry wastes glued with vegetal polyurethane adhesive: An efficient and eco-friendly alternative. Ind. Crops Prod..

[81] Jin X., Cui S., Zhang Y., Zhao X., Lv F., Sun S., Tian Y., Zhao Z., Liu D. (2022). Covalent bonding of the extruded polystyrene foams to mortar through ultraviolet-ozone irradiation. Constr. Build. Mater..

[82] (2015). Thermal Insulation Products for Buildings–Factory Made Wood Fibre (WF) Products–Specification.

[83] Mantanis G.I., Martinka J., Lykidis C., Ševčík L. (2019). Technological properties and fire performance of medium density fibreboard (MDF) treated with selected polyphosphate-based fire retardants. Wood Mater. Sci. Eng..

[84] Rodríguez G.E., Ávila C.B., Cloutier A. (2024). Physical and Mechanical Properties of Fiberboard Made of MDF Residues and Phase Change Materials. Forests.

